# Genetic tagging of the adenosine A2A receptor reveals its heterogeneous expression in brain regions

**DOI:** 10.3389/fnana.2022.978641

**Published:** 2022-08-18

**Authors:** Muran Wang, Zewen Li, Yue Song, Qiuqin Sun, Lu Deng, Zhiqing Lin, Yang Zeng, Chunhong Qiu, Jingjing Lin, Hui Guo, Jiangfan Chen, Wei Guo

**Affiliations:** ^1^The Molecular Neuropharmacology Laboratory and the Eye-Brain Research Center, The State Key Laboratory of Ophthalmology, Optometry and Vision Science, Wenzhou Medical University, Wenzhou, China; ^2^Department of Neurology, The Second Affiliated Hospital and Yuying Children’s Hospital of Wenzhou Medical University, Wenzhou, China; ^3^Key Laboratory of Structural Malformations in Children of Zhejiang Province, Wenzhou, China; ^4^Shanghai Pregen Biotechnology Co., Ltd., Shanghai, China

**Keywords:** adenosine A2A receptor (A_2A_R), knock-in mice, CRISPR/Cas9, striatum, lateral septum (LS), G protein-coupled receptor (GPCR)

## Abstract

The adenosine A_2A_ receptor (A_2A_R), a G protein-coupled receptor, is involved in numerous and varied physiological and pathological processes, including inflammation, immune responses, blood flow, and neurotransmission. Accordingly, it has become an important drug target for the treatment of neuropsychiatric disorders. However, the exact brain distribution of A_2A_R in regions outside the striatum that display relatively low levels of endogenous A_2A_R expression has hampered the exploration of A_2A_R functions under both physiological and pathological conditions. To further study the detailed distribution of the A_2A_R in low-expression regions, we have generated A_2A_R knock-in mice in which the 3xHA-2xMyc epitope tag sequence was fused to the C-terminus of A_2A_R (A_2A_R-tag mice) *via* CRISPR/Cas9 technology. Here, using CRISPR/Cas9 technology, we have generated A_2A_R knock-in mice in which the 3xHA-2xMyc epitope tag sequence was fused to the C-terminus of A_2A_R (A_2A_R-tag mice). The A_2A_R-tag mice exhibited normal locomotor activity and emotional state. Consistent with previous studies, A_2A_R fluorescence was widely detected in the striatum, nucleus accumbens, and olfactory tubercles, with numerous labeled cells being evident in these regions in the A_2A_R-tag mouse. Importantly, we also identified the presence of a few but clearly labeled cells in heterogeneous brain regions where A_2A_R expression has not previously been unambiguously detected, including the lateral septum, hippocampus, amygdala, cerebral cortex, and gigantocellular reticular nucleus. The A_2A_R-tag mouse represents a novel useful genetic tool for monitoring the expression of A_2A_R and dissecting its functions in brain regions other than the striatum.

## Introduction

The adenosine A_2A_ receptor (A_2A_R) is a class G protein-coupled receptor (GPCR) with key roles in the regulation of inflammation, immune responses, blood flow, and neurotransmission (de Lera Ruiz et al., 2014). Given these important functions, A_2A_R has attracted wide interest as a target for new drug development. In 2008, the A_2A_R agonist regadenoson was approved by the United States Food and Drug Administration (FDA) for use as a pharmacologic stress agent in myocardial reperfusion imaging (Chen et al., 2013). Meanwhile, in 2019, the US FDA also approved another A_2A_R antagonist, istradefylline, as an add-on treatment to levodopa for Parkinson’s disease (PD) “OFF” episodes (Chen and Cunha, 2020). Other candidates are in clinical trials as anti-hypertensives, anti-inflammatory compounds (Baraldi et al., 2018), or treatments for cancer (Vijayan et al., 2017).

In the brain, A_2A_Rs were initially thought to be exclusively present in basal ganglia, where they participate in the regulation of movement (Chen et al., 2013), sleep (Lazarus et al., 2012; Oishi et al., 2017; Tsai et al., 2021), and cognition (Chen and Cunha, 2020). However, subsequent investigations involving a variety of methods have provided conclusive evidence for the presence of A_2A_Rs in the limbic system and neocortex, albeit at a considerably lower density than in the striatum (Rosin et al., 1998; Lopes et al., 2004; Rebola et al., 2005a). Importantly, however, this low extra-striatal A_2A_R density does not imply that extra-striatal A_2A_Rs do not play an important role, as best exemplified by recent studies involving the hippocampus and amygdala. Stress can induce the upregulation of A_2A_Rs in the hippocampus (Batalha et al., 2013) or hippocampal nerve terminals (Kaster et al., 2015). A_2A_R blockade was reported to be efficient in reverting the behavioral and electrophysiological and morphological impairments induced by maternal separation, and this effect was associated with the restoration of the activity of the hypothalamic–pituitary–adrenal axis (Batalha et al., 2013). A three-week treatment with the A_2A_R antagonist SCH58261 reversed the mood and synaptic dysfunction resulting from exposure to chronic unpredictable stress (Kaster et al., 2015). In the amygdala, A_2A_Rs are enriched in glutamatergic synapses, where they selectively control synaptic plasticity at a major afferent pathway to this brain region. Notably, the downregulation of A_2A_Rs was shown to impair fear memory acquisition as well as fear memory retrieval in Pavlovian conditioning (Simoes et al., 2016). These observations highlight the need for a detailed determination of the expression pattern of this receptor to better understand its role in low-expression regions as well as identify the associated mechanism.

To monitor the expression of endogenous GPCRs, especially those with low abundance in the brain and for which specific antibodies are not yet available, mice expressing reporter genes such as GFP, luciferase, or β-galactosidase inserted downstream of the promoters of specific GPCRs have been generated (Ceredig and Massotte, 2015; Degrandmaison et al., 2022). Specifically, Lee et al. (2003) have generated mice harboring a construct consisting of a 4.8-kb promoter-proximal DNA fragment of the rat *Adora2a* gene fused to the lacZ coding sequence. LacZ was found to be expressed in many brain areas where functional A_2A_R is known to be expressed; however, LacZ expression was not observed to differ between the striatum and other areas of the brain. Recently, another strategy, A_2A_R-Cre mice mating with a ROSA26-EGFP reporter mice (Durieux et al., 2009), is employed to show the expression pattern of A_2A_R in the brain. To the best of our knowledge, the strategy has shown the highly enriched pattern of A_2A_R expression in striatopallidal neurons, however, whether this A_2A_R-Cre approach can be used to assess the low expression pattern of the A_2A_R outside the striatum is not clear. Furthermore, the use of A_2A_R-Cre line for expression pattern analysis would require additional breeding.

In the present study, we generated a novel mouse line in which the 3xHA-2xMyc epitope tag sequence was fused to the C-terminus of the A_2A_ receptor (A_2A_R-tag mice). We found that A_2A_R was highly expressed in the striatum, nucleus accumbens, and olfactory tubercles, consistent with that previously reported, and scarcely expressed in other regions, including the lateral septum (LS), hippocampus, amygdala, cerebral cortex, and gigantocellular reticular nucleus (Gi). The A_2A_R-tag mouse represents a powerful tool for determining the detailed expression pattern of A_2A_R as well as for the future immunoisolation of this receptor.

## Materials and methods

### Generation of A_2A_R-tag knock-in mice

In this study, CRISPR/Cas9 technology was employed to modify the mouse *Adora2a* gene. Briefly, Cas9 mRNA was transcribed *in vitro* as previously described (Yoshimi et al., 2014). The gRNAs were designed using the CRISPOR web tool^1^ to predict unique target sites throughout the mouse genome and transcribed *in vitro* using a T7 High Yield RNA Transcription Kit (Vazyme, Nanjing, China) according to the manufacturer’s instructions. The donor vector was constructed *via* In-fusion cloning. Fragments of left and right homologous arms flanking 3xHA-2xMyc for insertion were amplified using nested PCR. Nested PCR was also utilized to add 20-bp homologous adaptors to the junction sites of each homologous arm. The 3xHA-2xMyc fragment was synthesized by Genewiz (Suzhou, China). The three fragments were cloned together into a linearized HP361 vector by In-fusion cloning according to the manufacturer’s instructions. Cas9 mRNA, sgRNA, and the donor vector were microinjected into fertilized eggs of C57BL/6J mice. The eggs were then transplanted and F0 generation mice were identified by PCR and sequencing. A stable F1 generation was obtained by mating F0 mice positive for the construct with wild-type C57BL/6J mice. Heterozygous animals were intercrossed to generate A_2A_R-tag mice that were fertile and developed normally.

Male and female A_2A_-tag heterozygous mice or their wild-type littermates weighing 20–25 g were used for the subsequent analysis of A_2A_R expression. The animals were group-housed under standard laboratory conditions and kept on a 12-h day/12-h night cycle (lights on at 08:00 h). Mice were maintained and used in accordance with protocols approved by the Institutional Ethics Committee for Animal Use in Research and Education at Wenzhou Medical University, China.

### Mouse genotyping

Total DNA was isolated from the mouse tail using the DNeasy Blood & Tissue Kit (Qiagen, Hilden, Germany). PCR was performed at an annealing temperature of 60°C using the GoTaq^®^ Flexi DNA Kit (Promega, United States). The sequences of the primers used for genotyping were 5′-AGACCTTCCGGAAGATCATCCGA-3′ (forward) and 5′-TGGGGAGAGTAGTGTATTAGCAGG-3′ (reverse).

### Immunofluorescence staining

Six-to-eight-week-old male A_2A_R-tag C57BL/6J mice were anesthetized and perfused first with PBS and then with 4% PFA in PBS (pH 7.4). The brains were quickly removed, post-fixed in 4% PFA overnight, and then cryoprotected in 30% sucrose solutions in PBS for 3 days. The brain was cut into 30-μm-thick sections using a microtome (Leica CM1950) and preserved in PBS. Free-floating sections were blocked in blocking solution (0.3% Triton X-100 in PBS and 5% normal donkey serum) for 1 h at room temperature, incubated with the primary antibody in antibody solution (3% normal donkey serum, 0.3% Triton X-100 in PBS) overnight at 4°C, washed with PBS (3 × 10 min), incubated for 2 h at room temperature with the secondary antibodies (1: 500, No. A-11003, Invitrogen) and DAPI (No.C1006, Beyotime, China), and finally washed with PBS (3 × 10 min). The following antibodies were used: anti-HA antibody (1: 200, No.3724, Cell Signaling Technology, United States), anti-NeuN antibody (1: 1,000, No. MAB377, EMD Millipore, United States) and anti-c-Myc antibody (1:50, 1:200 or 1:500, No. 9E10, Santa Cruz Biotechnology, United States). Images were acquired with a Leica DM6B microscope or a Zeiss LSM 880 NLO confocal microscope.

### Primary cultures

The striatum was dissected from mouse pups (P0) and digested using papain. Cells were plated on poly-D-lysine-coated glass coverslips and cultured in B27/neurobasal A medium (Life Technologies, United States) containing 0.5 mM glutamine and 5 ng/ml basic fibroblast growth factor (Life Technologies). Fully matured primary neurons (6 days in culture) were used for immunofluorescence staining. Cells were fixed in 4% PFA for 15 min at room temperature, blocked (10% normal donkey serum and 0.3% Triton X-100 in PBS) for 1 h at room temperature, and then incubated with rabbit anti-HA antibody. After extensive washing with PBS containing 10% normal donkey serum and 0.3% Triton X-100, the cells were incubated with Alexa Fluor 488-conjugated donkey anti-rabbit IgG. Images were acquired with a Zeiss LSM 880 NLO confocal microscope.

### Behavioral tests

All behavior tests were performed in a sound-attenuated booth and were recorded on videotape for offline analysis. The animals were handled three times before the experiment. In all behavioral experiments, the experimenter was blinded to the genotype and/or treatment history.

#### Open field test

Animals were individually placed in the center of a chamber (40 cm × 40 cm × 40 cm) in a soundproof environment with gentle light for 10 min. The movement of each mouse was recorded and analyzed using the EthoVision XT system. Between trials, the chamber was cleaned with 10% ethanol.

#### Tail suspension test

The tail of each mouse was wrapped with tape approximately 1 cm from the end of the tail. Each mouse was then fixed upside down on a horizontal bar with the nose tip approximately 30 cm above the ground. Animal behaviors were recorded for 6 min from the side using the EthoVision XT system. Immobility time during the last 4 min was assessed offline by an observer blinded to the treatment.

#### Elevated zero maze test

The maze consisted of a circular track 5.5 cm wide, 46 cm in diameter, and raised 40 cm above the floor. The maze was divided into four quadrants of equal length with two opposing open quadrants with 1 cm high curbs to prevent falls and two opposing closed quadrants with 28-cm-high walls. A 10-min trial under gentle light conditions began when an animal was placed in the center of a closed quadrant. The movement of each mouse was recorded and analyzed using the EthoVision XT system. Between trials, the maze was cleaned with 10% ethanol.

### RNA extraction and real-time quantitative PCR

Striatum samples (12–16 mg/mouse) were collected from A_2A_R-tag (*n* = 6) or wild-type (*n* = 6) mice by microsurgical forceps under a stereomicroscope (Nikon, C-FLED2). Each sample was transferred into 1.5 ml EP tubes and immediately snap-frozen in liquid nitrogen. Then all samples were stored at –80°C for further use. For RT-qPCR experiment, each sample was lysed by 800 μl Trizol reagent (Invitrogen) and homogenized with a cryogenic freezing lapping machine (–20°C, 60 s, 60 Hz, 3 times, JXFSTPRP-CL, Shanghai Jingxin Industrial Development Co., Ltd., China). Then 160 μl chloroform was added for extraction. After 15 min reacting at room temperature and centrifugation for 15 min at 4°C with a super-centrifuge (13,000 rpm, Eppendorf 5810R), the supernatant was collected. The equivalent volume of isopropanol was added to the supernatant. After 10 min standing, the liquid was centrifugated for 10 min (4°C, 13,000 rpm), then the supernatant was discarded. The precipitation was washed with 75% ethanol (500 μl/sample) and dried in the fume hood. Finally, 40 μl RNase-free dH2O were added in each tube and mixed well. The RNA quality and concentration were assessed by NanoDrop 1000 3.8.1 (A260/A280 > 1.7; A260/A230 > 0.3). Reverse transcription was performed on 1,000 ng RNA and carried out using PrimeScript RT Master Mix (Takara, Japan) in 20 μl reaction mixtures. The reaction mixture was incubated first at 25°C for 10 min, and then at 37°C for 50 min, followed by heat inactivation at 70°C for 15 min. RT-qPCR analysis was carried out on the CFX96™ Optics Module using iTaq Universal SYBR Green Supermix (Bio-RAD, United States) (0.8 μl primer + 1.6 μl 1/20 dilutions of total cDNA). The thermal cycling program: 3 min at 95°C, 40 cycles of a two-step PCR, 95°C for 10 s followed by 55°C for 30 s, and a final extension from 65 to 95°C for 5 s. Relative A_2A_R expression levels were calculated by Livak method. The primers used for qPCR were: A_2A_R Forward: 5′-CCGAATTCCACTCCGGTACA-3′ and Reverse: 5′-CAGTTGTTCCAGCCCAGCAT-3′; GAPDH Forward: 5′-TTGTGATGGGTGTGAACCACGAGA-3′ and Reverse: 5′-GAGCCCTTCCACAATGCCAAAGTT-3′.

### Statistical analyses

All data were assessed for normality and homogeneity of variance. An unpaired Student’s *t*-test or Mann–Whitney *U* test was used to compare means between two groups. *P*-values <0.05 were considered significant. All data are presented as means ± SEM. No statistical methods were used to predetermine sample sizes; however, our sample sizes were similar to those normally employed in comparable studies.

## Results

### Generation of A_2A_R-tag knock-in mice

**FIGURE 1:**
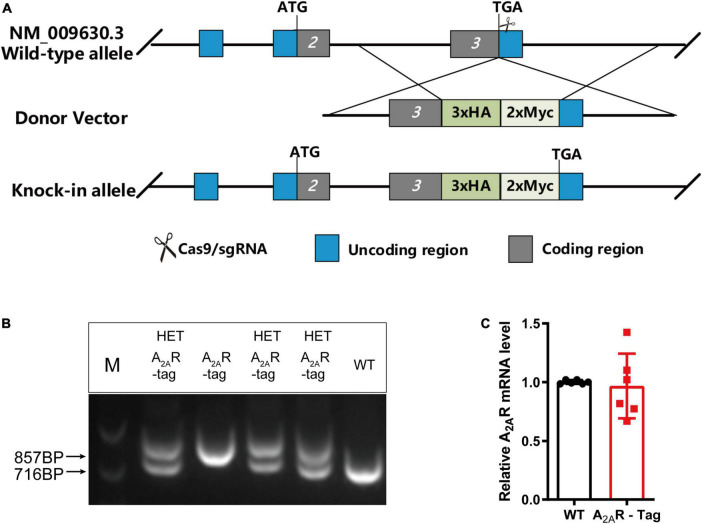
Knock-in construct, genotyping scheme, and adenosine A_2A_ receptor (A_2A_R) mRNA levels in A_2A_R-tag mice. **(A)** Targeting strategy. **(B)** PCR analysis of gene editing in A_2A_R-tag mice. Using tail genomic DNA, the 857-bp PCR product was only detected in A_2A_R-tag homozygous mice and the 716-bp product only in wild-type littermates, otherwise, two fragments (716-bp and 857-bp) from A_2A_R-tag heterozygous mice were detected. **(C)** The mRNA levels were determined by performing RT-PCR using striatum from A_2A_R-tag heterozygous mice, as described in “Materials and methods” section. *N* = 6 mice of each genotype. Data are mean ± SEM (each dot represents one mouse, Mann–Whitney *U* test).

In this study, we generated a novel transgenic mouse line in which the 3xHA-2xMyc epitope tag sequence YPYDVPD YA-YPYDVPDYA-YPYDVPDYA-EQKLISEEDL-EQKLISEEDL was fused to the C-terminus of A_2A_R using CRISPR/Cas9 technology (Figure 1A). Genotypes were confirmed by PCR using various primer combinations. These primers amplified a 857-bp fragment from A_2A_R-tag homozygous mice, a 716-bp fragment from wild-type littermates or two fragments (716-bp and 857-bp) from A_2A_R-tag heterozygous mice (Figure 1B). All A_2A_R-tag mice (homozygous or heterozygous) were viable and healthy and showed no differences in body weight compared with their wild-type siblings. In the present study, we used A_2A_R-tag heterozygous mice for further experiments, henceforth referred to as A_2A_R-tag mice. RT-qPCR was used to determine whether A_2A_R-tag mRNA expression levels differed from those of the endogenous receptor of wild-type mice. The expression levels of A_2A_R in the striatum of wild-type and A_2A_R-tag mice (*n* = 6 per genotype) were compared, and no significant differences in A_2A_R mRNA levels were detected between the two groups (Figure 1C). These results indicated that the genomic modification did not affect A_2A_R transcription.

**FIGURE 2:**
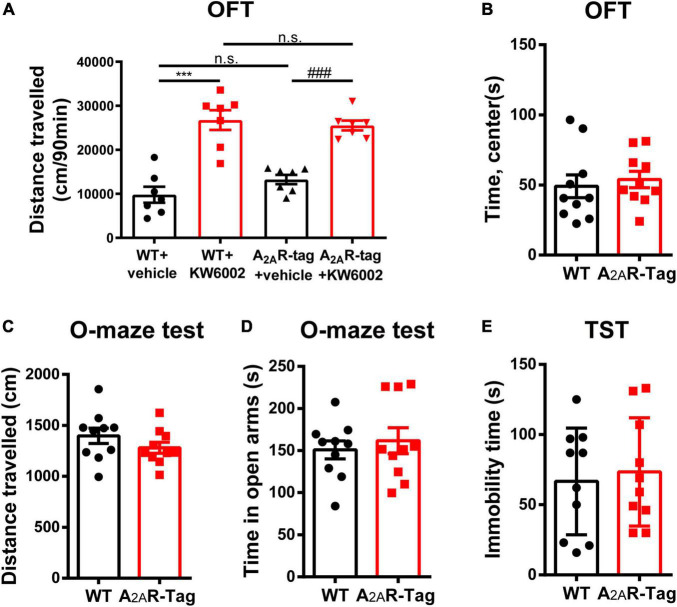
Adenosine A_2A_ receptor (A_2A_R)-tag mice showed no alternation in locomotor and mood tests. **(A)** After the intraperitoneal administration of KW6002 (5 mg/kg) or vehicle (DMSO + castor oil), mice were subjected to the open field test (OFT) for 90 min. The distances traveled by A_2A_R-tag mice or their wild-type littermates were significantly increased after KW6002 infusion, however, no difference in locomotor activity was observed between the two groups. Data are mean ± SEM (each dot represents one mouse, two-way ANOVA; interaction *P* > 0.05, KW6002; *P* < 0.0001; ***P<0.0001, ^###^*P*<0.0001, n.s. no significant). **(B)** Compared to the wild-type littermates, A_2A_R-tag mice showed no alternation of time spent in the center of open field. **(C,D)** Compared to the wild-type littermates, A_2A_R-tag mice displayed similar locomotion activity in elevated zero maze and spent a similar amount of time in the open arms. **(E)** A_2A_R-tag mice exhibited similar immobility time in the tail-suspension test (TST) compared with control animals. Data are mean ± SEM (each dot represents one mouse, unpaired Student’s *t*-test).

As A_2A_R is highly expressed in the striatum, an important region mediating movement and mood, the roles of the A_2A_R in locomotion and mood regulation have been extensively investigated in previous studies. A_2A_R antagonists increase motor activity in wild-type mice but its effect was abolished in A_2A_R KO mice in OFT (Shen et al., 2008). Similarly, the genetic deletion of A_2A_R attenuates maladaptive features in various anxiety or depressive-like behavioral paradigms (Yamada et al., 2013; Padilla et al., 2018). Accordingly, to assess the *in vivo* functional and physiological activation of A_2A_R in A_2A_R-tag mice, OFT, elevated zero maze test and TST were selected to assess the potential disruption of the A_2A_R pathway. In the present study, after the intraperitoneal administration of KW6002 (5 mg/kg) or vehicle (DMSO + castor oil), A_2A_R-tag mice were subjected to the OFT. The distance traveled by A_2A_R-tag mice was significantly increased after KW6002 infusion (Figure 2A). However, when A_2A_R-tag mice or their wild-type littermates all received KW6002 (5 mg/kg), no difference in locomotor activity was observed between the two groups (Figure 2A). And compare to the wild-type littermates, A_2A_R-tag mice showed no alternation of time spent in the center of open field (Figure 2B). Furthermore, to evaluate the basic mood state of A_2A_R-tag mice, the animals were subjected to the elevated zero maze test and the TST to evaluate their levels of anxiety or depression. Without change of locomotion activity in the elevated zero maze (Figure 2C), the A_2A_R-tag mice spent a similar amount of time in the open arms of an elevated zero maze (Figure 2D), and exhibited similar immobility time in the TST compared with control animals (Figure 2E).

### Anatomical profiling of A_2A_R distribution in A_2A_R-tag mice

**FIGURE 3:**
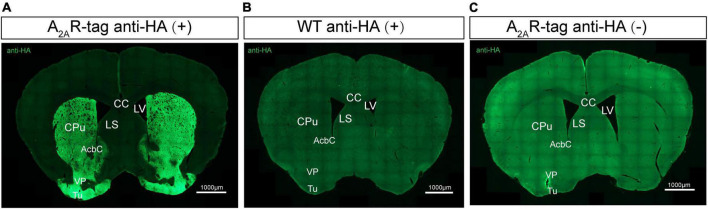
Evaluation of the specific HA expression in adult adenosine A_2A_ receptor (A_2A_R)-tag mice. **(A)** Coronal brain sections of A_2A_R-tag were incubated with an anti-HA antibody, and the most prominent and intense labeling was observed in the neuropil of the entire striatum and extended into the nucleus accumbens and olfactory tubercles. **(B)** There was no signal that could be detected in brain slices from wild-type mice after incubating with an anti-HA antibody. **(C)** Without adding an anti-HA antibody, there is no detectable staining signaling on brain slices from A_2A_R-tag mice. CPu, caudate putamen (striatum); AcbC, accumbens nucleus, core; VP, ventral pallidum; Tu, olfactory tubercles; LV, lateral ventricle; LS, lateral septal nucleus; CC, central canal. Scale bars as indicated in each picture.

Several studies have reported that A_2A_Rs are present in many regions of the brain (Rosin et al., 1998; Lopes et al., 2004; Rebola et al., 2005a; Chen et al., 2014). Here, we performed immunostaining on brain tissues using an anti-HA antibody to better understanding A_2A_R localization, focusing on areas where A_2A_R expression was previously reported to be low. Initial experiments demonstrated that anti-HA staining was detected only in A_2A_R-tag mice and not wild-type mice. The most prominent and intense labeling was observed in the neuropil of the entire striatum and extended into the nucleus accumbens and olfactory tubercles (Figures 3A,B). Additionally, no A_2A_R-tag signal could be detected in brain slices from A_2A_R-tag mice without the addition of the anti-HA antibody (Figure 3C). Similarly, the expression pattern of A_2A_R in A_2A_R-tag mice was assessed using anti-Myc antibody (No. 9E10, Santa Cruz Biotechnology). Unfortunately, no positive signal in the brain sections was detected (data not shown). Further verification must be needed *via* other anti-Myc antibodies.

**FIGURE 4:**
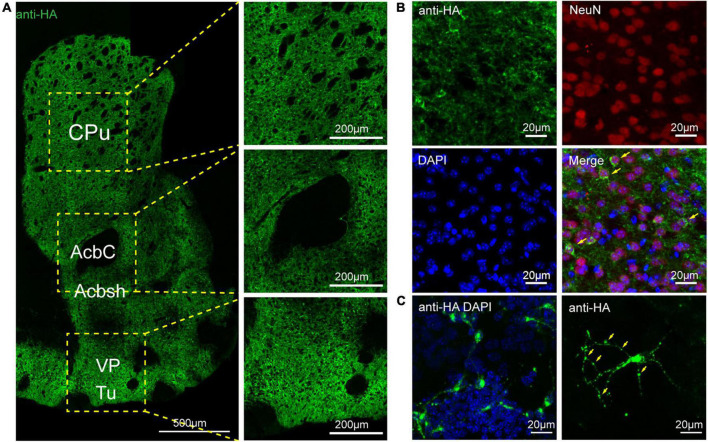
Distribution of HA immunoreactivity in the forebrain of adenosine A_2A_ receptor (A_2A_R)-tag mice. **(A, Left)** Representative confocal image of HA expression in the forebrain (1.18 mm before bregma). Dense labeling of the neuropil can be seen in the striatum, nucleus accumbens, ventral pallidum, and olfactory tubercles. **(Right)** High magnification of the areas in yellow indicated in panel **(A)**. Numerous labeled cells could be seen within the neuropil. **(B)** Double-labeling immunofluorescence experiment showed that anti-HA positive cells (green) were neurons (red) in the striatum. Notably, fluorescence seemed to be mostly localized to the plasma membrane (arrows), and the staining signal was denser in some areas at the cell membrane. **(C)** In fully mature primary neurons (6 days in culture) from A_2A_R-tag mouse, HA immunoreactivity displayed polarized localization in the cell body and a punctate pattern in the processes (arrows). CPu, caudate putamen (striatum); AcbC, accumbens nucleus, core; Acbsh, accumbens nucleus, shell; VP, ventral pallidum; Tu, olfactory tubercles. Scale bars as indicated in each picture.

**FIGURE 5:**
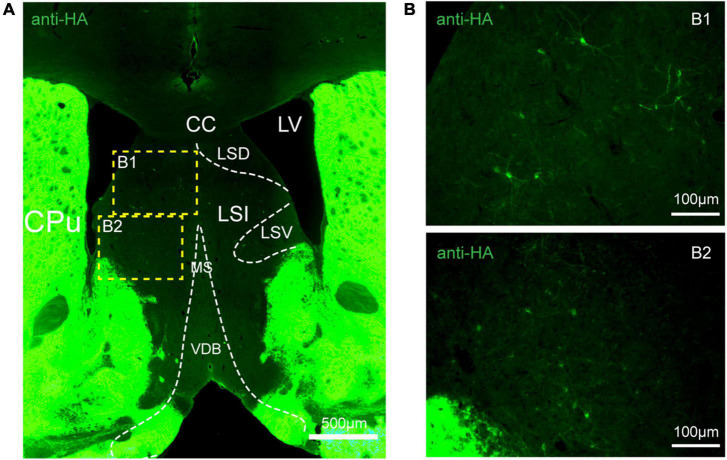
Distribution of HA immunoreactivity in the lateral septum of adenosine A_2A_ receptor (A_2A_R)-tag mice. **(A)** Representative confocal image of HA expression in the septum structure (0.86 mm before bregma). Bright labeled cells were observed in the LS and no staining was detected in the medial septum or the horizontal and vertical limbs of the diagonal band. **(B)** High magnification of the areas **(B1,B2)** in yellow indicated in panel **(A)**. CPu, caudate putamen (striatum); LV, lateral ventricle; CC, central canal; LSD, lateral septal nucleus, dorsal part; LSI, lateral septal nucleus, intermediate part; LSV, lateral septal nucleus, ventral part; MS, medial septal nucleus; VDB, nucleus of the vertical limb of the diagonal band. Scale bars as indicated in each picture.

**FIGURE 6:**
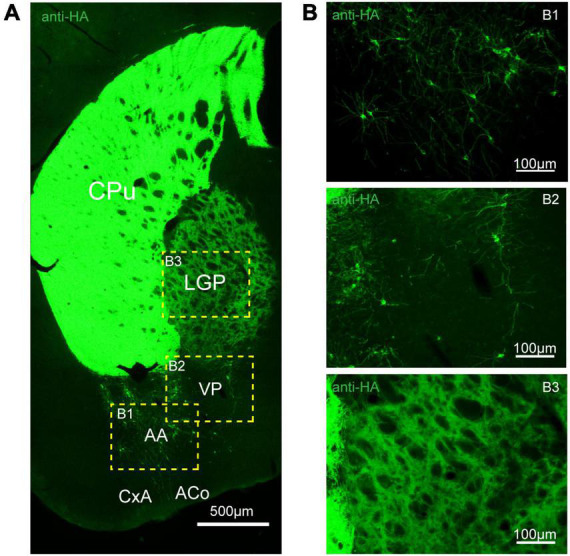
Distribution of HA immunoreactivity in the amygdala and globus pallidus of adenosine A_2A_ receptor (A_2A_R)-tag mice. **(A)** Representative confocal image of HA expression in the amygdala and globus pallidus (0.22 mm behind bregma). Discrete and bright labeled cells could be seen in the amygdala, mainly in the basolateral part. Immunoreactivity in the globus pallidus was lighter than in the striatum. **(B)** High magnification of the areas **(B1–B3)** in yellow indicated in panel **(A)**. CPu, caudate putamen (striatum); LGP, lateral globus pallidus; AA, anterior amygdaloid area; PV, ventral pallidum; CxA, cortex-amygdala transition zone; ACo, anterior cortical amygdaloid nucleus. Scale bars as indicated in each picture.

**FIGURE 7:**
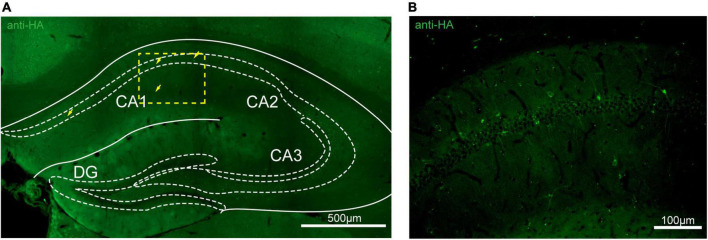
Distribution of HA immunoreactivity in the hippocampus of adenosine A_2A_ receptor (A_2A_R)-tag mice. **(A)** Representative confocal image of HA expression in the hippocampus (1.82 mm behind bregma). Very lightly labeled cells (arrows) with evident pyramidal morphology could be seen in the CA1 and CA2 regions, but not in the dentate gyrus. **(B)** High magnification of the area in yellow indicated in panel **(A)**. CA1, field CA1 of the hippocampus; CA2, field CA2 of hippocampus; CA3, field CA3 of the hippocampus; DG, dentate gyrus. Scale bars as indicated in each picture.

**FIGURE 8:**
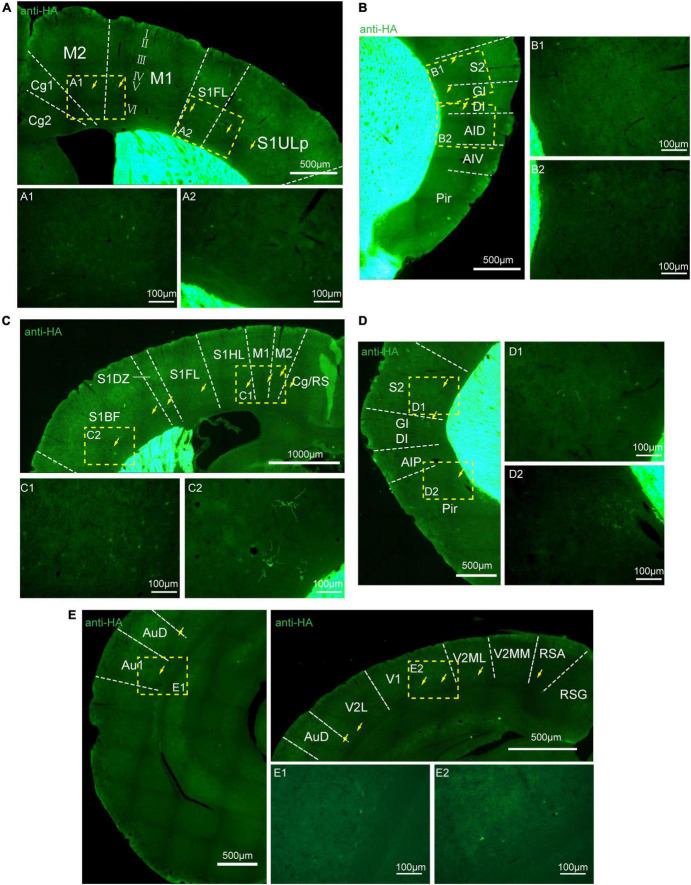
Distribution of HA immunoreactivity in the cerebral cortex of adenosine A_2A_ receptor (A_2A_R)-tag mice. **(A)** Up: representative confocal image of HA expression in the motor and somatosensory cortex (0.5 mm before bregma). Discrete and bright labeled cells (arrows) could be seen in the deep layers (layer V or VI). Down: high magnification of the areas **(A1,A2)** in yellow indicated in panel **(A)**. **(B, Left)** Representative confocal image of HA expression in the somatosensory and agranular insular cortex (0.5 mm before bregma). Discrete and bright labeled cells (arrows) could be seen in the deep layers (layer V or VI). **(Right)** High magnification of the areas **(B1,B2)** in yellow indicated in panel **(B)**. **(C, Up)** Representative confocal image of HA expression in the motor and somatosensory cortex (0.7 mm before bregma). Discrete and bright labeled cells (arrows) could be seen in the deep layers (layer V or VI). **(Down)** High magnification of the areas **(C1,C2)** in yellow indicated in panel **(C)**. **(D, Left)** Representative confocal image of HA expression in the somatosensory and agranular insular cortex (0.7 mm before bregma). Discrete and bright labeled cells (arrows) could be seen in the deep layers (layer V or VI). **(Right)** High magnification of the areas **(D1,D2)** in yellow indicated in panel **(D)**. **(E, Left and Right-up)** Representative confocal image of HA expression in the auditory and visual cortex (0.34 mm behind bregma). Discrete and bright labeled cells (arrows) could be seen in the deep layers (layer V or VI). **(Right-down)** High magnification of the areas **(E1,E2)** in yellow indicated in panel **(E)**. Cg, cingulate cortex, area 1; M2, secondary motor cortex; M1, primary motor cortex; S1FL, S1 cx, forelimb region; S1ULP, S1 cx, upper lip region; S2, secondary somatosensory cortex; GI, gigantocellular reticular nucleus; DI, dysgranular insular cortex; AID, agranular insular cortex, dorsal part; AIV, agranular insular cortex, ventral part; Pir,; RS, cingulate/retrosplenial; S1HL, S1 cx, hindlimb region; S1DZ, primary somatosensory cortex, dysgranular region; S1BF, S1 cx, barrel field; AIP, agranular insular cortex, posterior part; AuD, secondary auditory cortex, dorsal; Au1, primary auditory cortex; RSG, retrosplenial granular cortex; RSA, retrosplenial agranular cortex; V2MM, secondary vis cx, mediomed; V2ML, secondary visual cortex, mediolat; V1, primary visual cortex; V2L, secondary visual cortex, lateral area. Scale bars as indicated in each picture.

**FIGURE 9:**
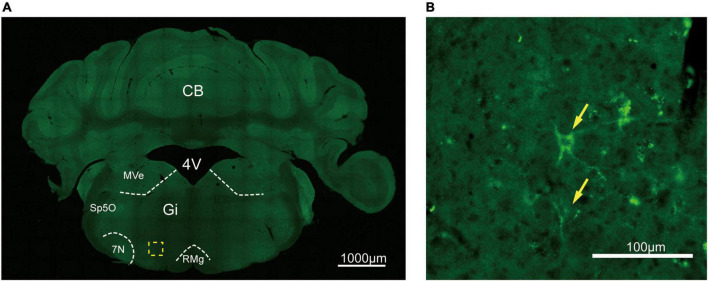
Distribution of HA immunoreactivity in the gigantocellular reticular nucleus of A_2A_R-tag mice. **(A)** Representative confocal image of HA expression in the brain (5.88 mm behind bregma). The labeled cell could be seen in the Gi. **(B)** High magnification of the area in yellow indicated in panel **(A)**. Discrete labeled cells (arrows) could be seen. CB, cerebellum; 4V, 4th ventricle; MVe, medial vestibular nucleus; Sp5O, spinal trigeminal nucleus, oral part; 7N, facial nucleus; Gi, gigantocellular reticular nucleus; RMg, raphe magnus nucleus. Scale bars as indicated in each picture.

To assess the distribution of the A_2A_R in the brain of A_2A_R-tag mouse, we examined coronal sections obtained at 120-μm intervals throughout the entire mouse brain using anti-HA antibody. A_2A_R was found to be highly expressed in the striatum, nucleus accumbens, and olfactory tubercles, where numerous labeled cells could be seen within the neuropil (Figure 4A). Double-labeling immunofluorescence of anti-HA positive cells in striatum revealed that A_2A_R mainly expressed in neurons. Notably, fluorescence seemed to be mostly localized to the plasma membrane (Figure 4B), and the staining signal was denser in some areas at the cell membrane. Recently, A_2A_Rs expression was detected in the axon initial segment, where it overlapped with voltage-gated Na^+^ channel (Nav) expression (Lezmy et al., 2021). This observation implied that A_2A_R expression is polarized in neurons. To confirm this possibility, primary neurons were prepared from striatum tissue dissected from A_2A_R-tag mouse pups (P0). In fully mature primary neurons (6 days in culture), A_2A_R immunoreactivity displayed polarized localization in the cell body and a punctate pattern in the processes (Figure 4C). Lightly labeled cells were observed in the LS and no staining was detected in the medial septum or the horizontal and vertical limbs of the diagonal band (Figure 5). A few discrete and bright labeled cells could be seen in the anterior amygdaloid area (Figures 6A,B1,B2). Immunoreactivity in the globus pallidus (Figures 6A,B3) was lighter than in the striatum. In the hippocampus, very lightly labeled cells with evident pyramidal morphology could be seen in the CA1 and CA2 regions, but not in the dentate gyrus (Figure 7). Additionally, a few discrete and bright labeled cells could be seen in the deep layers (layer V or VI) in certain areas of the cerebral cortex, including motor cortex, somatosensory, visual, insular and auditory cortex (Figure 8). Meanwhile, no signal was detected in the midbrain or cerebellum, and the Gi was the only area of the rhombencephalon with prominent labeling (Figure 9).

## Discussion

In the present study, using CRISPR/Cas9 technology, we generated a novel A_2A_R knock-in mouse line that will allow the study of A_2A_R in regions with relatively low levels of endogenous A_2A_R expression as well as the functions of A_2A_R under physiologically relevant conditions. We have previously generated A_2A_R knock-out (KO) mouse lines (Chen et al., 1999) and this “loss-of-function” approach has revolutionized the study of A_2A_R functions *in vivo* by offering a complementary approach to classical pharmacology. A_2A_R KO mice have been widely used for the investigation of stroke (Chen et al., 1999), sleep (Huang et al., 2005), PD (Chen et al., 2001; Fredduzzi et al., 2002), and cognition (Li Y. et al., 2015). Furthermore, the A_2A_R-Cre mouse line allows the precise targeting (Zhang et al., 2013) and manipulation of A_2A_R-expressing neurons (Yuan et al., 2017) *in vivo*. Recently, we have developed a chimeric rhodopsin–A_2A_R protein (optoA2AR) (Li P. et al., 2015) that contains the extracellular and transmembrane domains of rhodopsin (conferring light responsiveness and eliminating adenosine-binding pockets) fused to the intracellular loop of A_2A_R (to confer specific A_2A_R signaling), and this method has proved to be a powerful tool for the study of neuropsychiatric disorders (Li P. et al., 2015; Li Y. et al., 2015; Li et al., 2018). Despite significant advances, and like for other GPCRs, the study of A_2A_R in physiologically relevant conditions has been hampered by several challenges, such as relatively low endogenous expression levels (except in basal ganglia), the complexity of the transmembrane structure, and the lack of specific and potent antibodies (Michel et al., 2009; Jo and Jung, 2016). The development of new tools and a combination of approaches to study A_2A_R *in vivo* would be beneficial for the further understanding of the receptor and, consequently, also crucial for improving current A_2A_R-targeted therapeutics.

Recent investigations have shown the important roles of A_2A_R in the hippocampus and amygdala, where A_2A_R is expressed at low levels. Here, we have reported that A_2A_R is unambiguously expressed in other brain regions, although less abundantly, which merits further investigation. Notably, the role of A_2A_R in the LS is an interesting topic considering the important role of LS in the regulation of a variety of functions, including social aggression, locomotion, kinship, and anxiety (Besnard and Leroy, 2022; Sheehan et al., 2004). Furthermore, the functions of A_2A_R in the brainstem are largely undefined owing to the limited distribution of the receptor in such complex regions. In the present study, we found that A_2*A*_R is expressed in the Gi, which is the main output center for movement control (Brownstone and Chopek, 2018) with a significant role in locomotor recovery after incomplete spinal cord injury (Engmann et al., 2020). To explore the potential functions of A_2A_R in these regions, more detailed information about all aspects of the receptor is required, including knowledge of the cell types that express A_2A_R and the neural circuit connections involving A_2A_R-positive neurons. Achieving this goal requires the development of novel methods, such as Cre-dependent virus tracing, optogenetics, and designer receptors exclusively activated by designer drugs.

The cellular polarization of A_2A_R expression warrants further investigation. In our study, we found that A_2A_-tag receptor localization was polarized in the cell body in tissue slices and primary cultures derived from the striatum, while A_2A_R immunoreactivity displayed a punctate pattern in neuronal processes. This expression pattern is consistent with that of previous reports. In cultured hippocampal neurons, A_2*A*_R immunoreactivity displayed a punctate pattern and mostly co-localized with synaptophysin immunoreactivity, indicating that it was located at nerve terminals (Rebola et al., 2005a,b). The *in vitro* finding is supported by a further *in vivo* study that found the presynaptically localization of A_2A_Rs in nerve terminals in the hippocampus (Kaster et al., 2015). Given that polarized A_2*A*_R distribution may be involved in regulating myelinated axon excitability (Lezmy et al., 2021), it is important that additional studies are undertaken to further understand the role of this polarized distribution and identify the associated mechanisms.

Previous studies have demonstrated that A_2A_R are not only expressed in neurons but also in astrocytes in the brain (Matos et al., 2013, 2015) in the brain. Astrocytic A_2A_R is involved in varied functions under physiological conditions: triggering transcriptional deregulation (Paiva et al., 2019) and mediating astrocyte reactivity (Brambilla et al., 2003; Ke et al., 2009), controlling glutamate release and consequently synaptic transmission (Nishizaki et al., 2002; Cervetto et al., 2017), regulating glutamate uptake by controlling the levels of glutamate transporters and the activity of Na + /K + -ATPase (Nishizaki et al., 2002; Matos et al., 2013). However, these effects are merely observed in cultured astrocytes or gliosomes (Matos et al., 2013) *via* pharmacological methods as the low expression of astrocytic A_2A_R in physical conditions. In our A_2A_R-tag mouse, the HA, under the control of the A_2A_R endogenous promoter, must be expressed at similarly low levels in astrocytes. However, the findings (Angulo et al., 2003; Orr et al., 2015) that the increased level of A_2A_R in astrocytes in the brain of Alzheimer’s Disease patients raise the interesting to investigate the role of astrocytic A_2A_R under pathological conditions. The A_2A_R-tag mouse would represent a useful tool for further studies.

Our A_2A_R-tag mouse line also represents an excellent tool to examine the expression and distribution of A_2A_R *in vivo* during development. In the developing rat nervous system, widespread and partly transient distribution of A_2A_R has been found in several areas, including the cerebral cortex, subiculum, parafascicularis nucleus of the thalamus, facial nucleus, trigeminal nucleus, locus coeruleus, area postrema, anterior pituitary gland, and fetal cerebral vasculature (Weaver, 1993). There is also some evidence to support that A_2A_R plays a role in the regulation of the radial migration of cortical excitatory neurons as well as the tangential migration of interneurons (Silva et al., 2013). Recently, A_2A_R activation was reported to contribute to the migration of cortical projection neurons in the transition from the lower intermediate zone to the cortical plate by controlling their polarization and axon formation and outgrowth (Alcada-Morais et al., 2021). Some evidence also supports a role for adenosine as a fine-tuning regulator of the development of cortical cytoarchitecture, in particular in the integration of the wiring of excitatory and inhibitory cortical networks. However, to understand the long-term impact of this ability of A_2A_R to control the migration of both interneurons and principal neurons, strategies other than global KO mice are necessary, particularly those that allow the precise spatial–temporal regulation of A_2A_R function during embryogenesis or postnatally.

The particularly long (122 residues) C-terminus of A_2A_R is considered to be an important relay for A_2A_R interaction with other proteins (Nguyen et al., 2021). To exclude the possibility that the epitope tag might result in an abnormal interaction and leads to an altered phenotype, A_2A_R-tag mice were exposed to several behavioral tests to determine whether the modified receptor retained its *in vivo* endogenous function. We did not find any abnormalities in A_2A_R-tag mice, as previously reported for other tagged GPCRs. The C-terminal tagging of GPCRs with green fluorescent protein (GFP) is generally thought to have no significant impact on GPCR properties, including ligand binding, signal transduction, and intracellular trafficking (Ceredig and Massotte, 2015). Specifically, C-terminal GFP-tagged adenosine receptor A1Rs, A_2A_Rs, and A3Rs have been expressed in primary hippocampal neurons to demonstrate the differential trafficking of these receptors in neurons (Baines et al., 2011). Nevertheless, additional investigations are required to exclude the possibility of abnormal signaling transduction in our A_2A_R-tag mice.

Aberrant protein–protein interactions (PPIs) are associated with various pathological conditions, including cancer, infectious diseases, and neurodegenerative diseases. Accordingly, targeting PPIs holds promise in the treatment of these diseases and represents an essential strategy for the development of novel therapeutic drugs targeting these conditions (Lu et al., 2020). The list of A_2A_R-interacting partners is long and rapidly growing (Keuerleber et al., 2011), and includes translin-X-associated protein (Chien et al., 2018), heat-shock proteins (Bergmayr et al., 2013), GAS-2-like protein 2 (Wu et al., 2013), and calmodulin (Piirainen et al., 2015). Furthermore, A_2A_R can form a heteromeric complex with at least one other GPCR, namely, the D2 dopamine receptor, cannabinoid CB1 receptors (Carriba et al., 2007), fibroblast growth factor receptors (Flajolet et al., 2008), and subtype 5 metabotropic glutamate (mGluR5) (Ferre et al., 2002), and can also transactivate the neurotrophin receptors TrkA and TrkB (Wiese et al., 2007). Although much is already known about the expression and function of A_2A_R, most relevant studies were performed in heterologous systems overexpressing the receptor *in vitro*, and many proteins that interact with A_2A_R *in vivo* (under the control of its endogenous promoter) remain to be identified. Our findings showed that the A_2A_R-tag is expressed at similar levels and in the same brain areas as the wild-type receptor, which should allow us to identify endogenous A_2A_R-interacting proteins in the brain in future investigations (Degrandmaison et al., 2020).

In summary, the generation and initial characterization of the A_2A_R-tag knock-in mice will allow more detailed regional tissue distribution studies, co-localization investigations, and interactome analysis, as well as provide an impetus for an improvement in the understanding of the functions of A_2A_R *in vivo*.

## Data availability statement

The raw data supporting the conclusions of this article will be made available by the authors, without undue reservation.

## Ethics statement

The animal study was reviewed and approved by the Institutional Ethics Committee for Animal Use in Research and Education at Wenzhou Medical University.

## Author contributions

WG, MW, and ZWL: conceptualization. WG and MW: methodology. MW, ZWL, YS, QS, LD, and ZQL: formal analysis. MW, ZL, YS, QS, and LD: investigation. YZ, CQ, JL, and HG: resources. WG, MW, ZWL, and YZ: writing—original draft. WG and JC: writing—review and editing, funding acquisition. MW, ZWL, YS, and QS: visualization. JC: supervision. WG: project administration. All authors contributed to the article and approved the submitted version.

## Footnotes

1
http://crispor.tefor.net

## References

[B1] Alcada-Morais S. Goncalves N. Moreno-Juan V. Andres B. Ferreira S. Marques J. M. (2021). Adenosine A(2A) receptors contribute to the radial migration of cortical projection neurons through the regulation of neuronal polarization and axon formation. *Cereb. Cortex* 31 5652–5663. 10.1093/cercor/bhab188 34184030

[B2] Angulo E. Casadó V. Mallol J. Canela E. I. Viñals F. Ferrer I. (2003). A1 adenosine receptors accumulate in neurodegenerative structures in Alzheimer disease and mediate both amyloid precursor protein processing and tau phosphorylation and translocation. *Brain Pathol.* 13 440–451. 10.1111/j.1750-3639.2003.tb00475.x 14655750 PMC8095992

[B3] Baines A. E. Correa S. A. Irving A. J. Frenguelli B. G. (2011). Differential trafficking of adenosine receptors in hippocampal neurons monitored using GFP- and super-ecliptic pHluorin-tagged receptors. *Neuropharmacology* 61 1–11. 10.1016/j.neuropharm.2011.02.005 21315741

[B4] Baraldi S. Baraldi P. G. Oliva P. Toti K. S. Ciancetta A. Jacobson K. A. (2018). “A2A adenosine receptor: Structures, modeling, and medicinal chemistry,” in *The Adenosine Receptors*, ed. Borea P. Varani K. Gessi S. Merighi S. Vincenzi F. (Totowa, NJ: Humana Press), 91–136.

[B5] Batalha V. L. Pego J. M. Fontinha B. M. Costenla A. R. Valadas J. S. Baqi Y. (2013). Adenosine A(2A) receptor blockade reverts hippocampal stress-induced deficits and restores corticosterone circadian oscillation. *Mol. Psychiatry* 18 320–331. 10.1038/mp.2012.8 22371048

[B6] Bergmayr C. Thurner P. Keuerleber S. Kudlacek O. Nanoff C. Freissmuth M. (2013). Recruitment of a cytoplasmic chaperone relay by the A2A adenosine receptor. *J. Biol. Chem.* 288 28831–28844. 10.1074/jbc.M113.464776 23965991 PMC3789979

[B7] Besnard A. Leroy F. (2022). Top-down regulation of motivated behaviors via lateral septum sub-circuits. *Mol. Psychiatry* 10.1038/s41380-022-01599-3 [Epub ahead of print]. 35581296 PMC7613864

[B8] Brambilla R. Cottini L. Fumagalli M. Ceruti S. Abbracchio M. P. (2003). Blockade of A2A adenosine receptors prevents basic fibroblast growth factor-induced reactive astrogliosis in rat striatal primary astrocytes. *Glia* 43 190–194. 10.1002/glia.10243 12838511

[B9] Brownstone R. M. Chopek J. W. (2018). Reticulospinal systems for tuning motor commands. *Front. Neural Circuits* 12:30. 10.3389/fncir.2018.00030 29720934 PMC5915564

[B10] Carriba P. Ortiz O. Patkar K. Justinova Z. Stroik J. Themann A. (2007). Striatal adenosine A(2A) and Cannabinoid CB1 receptors form functional heteromeric complexes that mediate the motor effects of Cannabinoids. *Neuropsychopharmacology* 32 2249–2259. 10.1038/sj.npp.1301375 17356572

[B11] Ceredig R. A. Massotte D. (2015). Fluorescent knock-in mice to decipher the physiopathological role of G protein-coupled receptors. *Front. Pharmacol.* 5:289. 10.3389/fphar.2014.00289 25610398 PMC4284998

[B12] Cervetto C. Venturini A. Passalacqua M. Guidolin D. Genedani S. Fuxe K. (2017). A2A-D2 receptor-receptor interaction modulates gliotransmitter release from striatal astrocyte processes. *J. Neurochem.* 140 268–279. 10.1111/jnc.13885 27896809

[B13] Chen J. F. Cunha R. A. (2020). The belated US FDA approval of the adenosine A2A receptor antagonist istradefylline for treatment of Parkinson’s disease. *Purinergic Signal.* 16 167–174. 10.1007/s11302-020-09694-2 32236790 PMC7367999

[B14] Chen J. F. Eltzschig H. K. Fredholm B. B. (2013). Adenosine receptors as drug targets–what are the challenges? *Nat. Rev. Drug Discov.* 12 265–286. 10.1038/nrd3955 23535933 PMC3930074

[B15] Chen J. F. Huang Z. Ma J. Zhu J. Moratalla R. Standaert D. (1999). A(2A) adenosine receptor deficiency attenuates brain injury induced by transient focal ischemia in mice. *J. Neurosci.* 19 9192–9200. 10.1523/JNEUROSCI.19-21-09192.1999 10531422 PMC6782932

[B16] Chen J. F. Lee C. F. Chern Y. (2014). Adenosine receptor neurobiology: Overview. *Int. Rev. Neurobiol.* 119 1–49. 10.1016/B978-0-12-801022-8.00001-5 25175959

[B17] Chen J. F. Moratalla R. Impagnatiello F. Grandy D. K. Cuellar B. Rubinstein M. (2001). The role of the D(2) dopamine receptor (D(2)R) in A(2A) adenosine receptor (A(2A)R)-mediated behavioral and cellular responses as revealed by A(2A) and D(2) receptor knockout mice. *Proc. Natl. Acad. Sci. U.S.A.* 98 1970–1975. 10.1073/pnas.98.4.1970 11172060 PMC29366

[B18] Chien T. Weng Y. T. Chang S. Y. Lai H. L. Chiu F. L. Kuo H. C. (2018). GSK3beta negatively regulates TRAX, a scaffold protein implicated in mental disorders, for NHEJ-mediated DNA repair in neurons. *Mol. Psychiatry* 23 2375–2390. 10.1038/s41380-017-0007-z 29298990 PMC6294740

[B19] de Lera Ruiz M. Lim Y. H. Zheng J. (2014). Adenosine A2A receptor as a drug discovery target. *J. Med. Chem.* 57 3623–3650. 10.1021/jm4011669 24164628

[B20] Degrandmaison J. Abdallah K. Blais V. Genier S. Lalumiere M. P. Bergeron F. (2020). *In vivo* mapping of a GPCR interactome using knockin mice. *Proc. Natl. Acad. Sci. U.S.A.* 117 13105–13116. 10.1073/pnas.1917906117 32457152 PMC7293596

[B21] Degrandmaison J. Rochon-Hache S. Parent J. L. Gendron L. (2022). Knock-in mouse models to investigate the functions of opioid receptors *in vivo*. *Front. Cell Neurosci.* 16:807549. 10.3389/fncel.2022.807549 35173584 PMC8841419

[B22] Durieux P. F. Bearzatto B. Guiducci S. Buch T. Waisman A. Zoli M. (2009). D2R striatopallidal neurons inhibit both locomotor and drug reward processes. *Nat. Neurosci.* 12 393–395. 10.1038/nn.2286 19270687

[B23] Engmann A. K. Bizzozzero F. Schneider M. P. Pfyffer D. Imobersteg S. Schneider R. (2020). The gigantocellular reticular nucleus plays a significant role in locomotor recovery after incomplete spinal cord injury. *J. Neurosci.* 40 8292–8305. 10.1523/JNEUROSCI.0474-20.2020 32978289 PMC7577599

[B24] Ferre S. Karcz-Kubicha M. Hope B. T. Popoli P. Burgueno J. Gutierrez M. A. (2002). Synergistic interaction between adenosine A2A and glutamate mGlu5 receptors: Implications for striatal neuronal function. *Proc. Natl. Acad. Sci. U.S.A.* 99 11940–11945. 10.1073/pnas.172393799 12189203 PMC129373

[B25] Flajolet M. Wang Z. Futter M. Shen W. Nuangchamnong N. Bendor J. (2008). FGF acts as a co-transmitter through adenosine A(2A) receptor to regulate synaptic plasticity. *Nat. Neurosci.* 11 1402–1409. 10.1038/nn.2216 18953346 PMC2779562

[B26] Fredduzzi S. Moratalla R. Monopoli A. Cuellar B. Xu K. Ongini E. (2002). Persistent behavioral sensitization to chronic L-DOPA requires A2A adenosine receptors. *J. Neurosci.* 22 1054–1062. 10.1523/JNEUROSCI.22-03-01054.2002 11826134 PMC6758487

[B27] Huang Z. L. Qu W. M. Eguchi N. Chen J. F. Schwarzschild M. A. Fredholm B. B. (2005). Adenosine A2A, but not A1, receptors mediate the arousal effect of caffeine. *Nat. Neurosci.* 8 858–859. 10.1038/nn1491 15965471

[B28] Jo M. Jung S. T. (2016). Engineering therapeutic antibodies targeting G-protein-coupled receptors. *Exp. Mol. Med.* 48:e207. 10.1038/emm.2015.105 26846450 PMC4892866

[B29] Kaster M. P. Machado N. J. Silva H. B. Nunes A. Ardais A. P. Santana M. (2015). Caffeine acts through neuronal adenosine A2A receptors to prevent mood and memory dysfunction triggered by chronic stress. *Proc. Natl. Acad Sci. U.S.A.* 112 7833–7838. 10.1073/pnas.1423088112 26056314 PMC4485143

[B30] Ke R. H. Xiong J. Liu Y. Ye Z. R. (2009). Adenosine A2A receptor induced gliosis *via* Akt/NF-kappaB pathway *in vitro*. *Neurosci. Res.* 65 280–285. 19666061 10.1016/j.neures.2009.08.002

[B31] Keuerleber S. Gsandtner I. Freissmuth M. (2011). From cradle to twilight: The carboxyl terminus directs the fate of the A(2A)-adenosine receptor. *Biochim. Biophys. Acta* 1808 1350–1357. 10.1016/j.bbamem.2010.05.009 20478264

[B32] Lazarus M. Huang Z. L. Lu J. Urade Y. Chen J. F. (2012). How do the basal ganglia regulate sleep-wake behavior? *Trends Neurosci.* 35 723–732. 10.1016/j.tins.2012.07.001 22858523

[B33] Lee Y. C. Chien C. L. Sun C. N. Huang C. L. Huang N. K. Chiang M. C. (2003). Characterization of the rat A2A adenosine receptor gene: A 4.8-kb promoter-proximal DNA fragment confers selective expression in the central nervous system. *Eur. J. Neurosci.* 18 1786–1796. 10.1046/j.1460-9568.2003.02907.x 14622213

[B34] Lezmy J. I Arancibia-Carcamo L. Quintela-Lopez T. Sherman D. L. Brophy P. J. Attwell D. (2021). Astrocyte Ca(2+)-evoked ATP release regulates myelinated axon excitability and conduction speed. *Science* 374:eabh2858. 10.1126/science.abh2858 34648330 PMC7611967

[B35] Li P. Rial D. Canas P. M. Yoo J. H. Li W. Zhou X. (2015). Optogenetic activation of intracellular adenosine A2A receptor signaling in the hippocampus is sufficient to trigger CREB phosphorylation and impair memory. *Mol. Psychiatry* 20 1339–1349. 10.1038/mp.2014.182 25687775 PMC4539301

[B36] Li Y. He Y. Chen M. Pu Z. Chen L. Li P. (2015). Optogenetic activation of adenosine A2A receptor signaling in the dorsomedial striatopallidal neurons suppresses goal-directed behavior. *Neuropsychopharmacology* 41 1003–1013. 10.1038/npp.2015.227 26216520 PMC4748425

[B37] Li Z. Chen X. Wang T. Gao Y. Li F. Chen L. (2018). The corticostriatal adenosine A2A receptor controls maintenance and retrieval of spatial working memory. *Biol. Psychiatry* 83 530–541. 10.1016/j.biopsych.2017.07.017 28941549

[B38] Lopes L. V. Halldner L. Rebola N. Johansson B. Ledent C. Chen J. F. (2004). Binding of the prototypical adenosine A(2A) receptor agonist CGS 21680 to the cerebral cortex of adenosine A(1) and A(2A) receptor knockout mice. *Br. J. Pharmacol.* 141 1006–1014. 10.1038/sj.bjp.0705692 14993095 PMC1574266

[B39] Lu H. Zhou Q. He J. Jiang Z. Peng C. Tong R. (2020). Recent advances in the development of protein-protein interactions modulators: Mechanisms and clinical trials. *Signal Transduct. Target. Ther.* 5:213. 10.1038/s41392-020-00315-3 32968059 PMC7511340

[B40] Matos M. Augusto E. Agostinho P. Cunha R. A. Chen J. F. (2013). Antagonistic interaction between adenosine A2A receptors and Na+/K+-ATPase-α2 controlling glutamate uptake in astrocytes. *J. Neurosci.* 33 18492–18502. 10.1523/JNEUROSCI.1828-13.2013 24259572 PMC3834055

[B41] Matos M. Shen H. Y. Augusto E. Wang Y. Wei C. J. Wang Y. T. (2015). Deletion of adenosine A2A receptors from astrocytes disrupts glutamate homeostasis leading to psychomotor and cognitive impairment: Relevance to schizophrenia. *Biol. Psychiatry* 78 763–774. 10.1016/j.biopsych.2015.02.026 25869810 PMC4714966

[B42] Michel M. C. Wieland T. Tsujimoto G. (2009). How reliable are G-protein-coupled receptor antibodies? *Naunyn Schmiedebergs Arch. Pharmacol.* 379 385–388. 10.1007/s00210-009-0395-y 19172248

[B43] Nguyen K. D. Q. Vigers M. Sefah E. Seppala S. Hoover J. P. Schonenbach N. S. (2021). Homo-oligomerization of the human adenosine A2A receptor is driven by the intrinsically disordered C-terminus. *Elife* 10:e66662. 10.7554/eLife.66662 34269678 PMC8328514

[B44] Nishizaki T. Nagai K. Nomura T. Tada H. Kanno T. Tozaki H. (2002). A new neuromodulatory pathway with a glial contribution mediated *via* A(2a) adenosine receptors. *Glia* 39 133–147. 10.1002/glia.10100 12112365

[B45] Oishi Y. Xu Q. Wang L. Zhang B. J. Takahashi K. Takata Y. (2017). Slow-wave sleep is controlled by a subset of nucleus accumbens core neurons in mice. *Nat. Commun.* 8:734. 10.1038/s41467-017-00781-4 28963505 PMC5622037

[B46] Orr A. G. Hsiao E. C. Wang M. M. Ho K. Kim D. H. Wang X. (2015). Astrocytic adenosine receptor A2A and Gs-coupled signaling regulate memory. *Nat. Neurosci.* 18 423–434. 10.1038/nn.3930 25622143 PMC4340760

[B47] Padilla K. M. Quintanar-Setephano A. ópez-Vallejo F. L. Berumen L. C. Miledi R. García-Alcocer G. (2018). Behavioral changes induced through adenosine A2A receptor ligands in a rat depression model induced by olfactory bulbectomy. *Brain Behav.* 8:e00952. 10.1002/brb3.952 29761007 PMC5943772

[B48] Paiva I. Carvalho K. Santos P. Cellai L. Pavlou M. A. S. Jain G. (2019). A(2A) R-induced transcriptional deregulation in astrocytes: An *in vitro* study. *Glia* 67 2329–2342. 10.1002/glia.23688 31328322

[B49] Piirainen H. Hellman M. Tossavainen H. Permi P. Kursula P. Jaakola V. P. (2015). Human adenosine A2A receptor binds calmodulin with high affinity in a calcium-dependent manner. *Biophys. J.* 108 903–917. 10.1016/j.bpj.2014.12.036 25692595 PMC4336363

[B50] Rebola N. Canas P. M. Oliveira C. R. Cunha R. A. (2005a). Different synaptic and subsynaptic localization of adenosine A2A receptors in the hippocampus and striatum of the rat. *Neuroscience* 132 893–903. 10.1016/j.neuroscience.2005.01.014 15857695

[B51] Rebola N. Rodrigues R. J. Oliveira C. R. Cunha R. A. (2005b). Different roles of adenosine A1, A2A and A3 receptors in controlling kainate-induced toxicity in cortical cultured neurons. *Neurochem. Int.* 47 317–325. 10.1016/j.neuint.2005.05.009 16011860

[B52] Rosin D. L. Robeva A. Woodard R. L. Guyenet P. G. Linden J. (1998). Immunohistochemical localization of adenosine A2A receptors in the rat central nervous system. *J. Comp. Neurol.* 401 163–186. 10.1002/(SICI)1096-9861(19981116)401:2<163::AID-CNE2>3.0.CO;2-D 9822147

[B53] Sheehan T. P. Chambers R. A. Russell D. S. (2004). Regulation of affect by the lateral septum: implications for neuropsychiatry. *Brain Res. Brain Res. Rev.* 46, 71–117. 15297155 10.1016/j.brainresrev.2004.04.009

[B54] Shen H. Y. Coelho J. E. Ohtsuka N. Canas P. M. Day Y. J. Huang Q. Y. (2008). A critical role of the adenosine A2A receptor in extrastriatal neurons in modulating psychomotor activity as revealed by opposite phenotypes of striatum and forebrain A2A receptor knock-outs. *J. Neurosci.* 28 2970–2975. 10.1523/JNEUROSCI.5255-07.2008 18354001 PMC6670718

[B55] Silva C. G. Metin C. Fazeli W. Machado N. J. Darmopil S. Launay P. S. (2013). Adenosine receptor antagonists including caffeine alter fetal brain development in mice. *Sci. Transl. Med.* 5:197ra104. 10.1126/scitranslmed.3006258 23926202

[B56] Simoes A. P. Machado N. J. Goncalves N. Kaster M. P. Simoes A. T. Nunes A. (2016). Adenosine A2A receptors in the amygdala control synaptic plasticity and contextual fear memory. *Neuropsychopharmacology* 41 2862–2871. 10.1038/npp.2016.98 27312408 PMC5061896

[B57] Tsai C. J. Nagata T. Liu C. Y. Suganuma T. Kanda T. Miyazaki T. (2021). Cerebral capillary blood flow upsurge during REM sleep is mediated by A2A receptors. *Cell Rep.* 36:109558. 10.1016/j.celrep.2021.109558 34407410

[B58] Vijayan D. Young A. Teng M. W. L. Smyth M. J. (2017). Targeting immunosuppressive adenosine in cancer. *Nat. Rev. Cancer.* 17 709–724. 10.1038/nrc.2017.86 29059149

[B59] Weaver D. R. (1993). A2A adenosine receptor gene expression in developing rat brain. *Brain Res. Mol. Brain Res.* 20 313–327. 8114618 10.1016/0169-328x(93)90058-w

[B60] Wiese S. Jablonka S. Holtmann B. Orel N. Rajagopal R. Chao M. V. (2007). Adenosine receptor A2A-R contributes to motoneuron survival by transactivating the tyrosine kinase receptor TrkB. *Proc. Natl. Acad. Sci. U.S.A.* 104 17210–17215. 10.1073/pnas.0705267104 17940030 PMC2040418

[B61] Wu Y. C. Lai H. L. Chang W. C. Lin J. T. Liu Y. J. Chern Y. (2013). A novel Gαs-binding protein, Gas-2 like 2, facilitates the signaling of the A2A adenosine receptor. *Biochim. Biophys. Acta* 1833 3145–3154. 10.1016/j.bbamcr.2013.08.009 23994616

[B62] Yamada K. Kobayashi M. Mori A. Jenner P. Kanda T. (2013). Antidepressant-like activity of the adenosine A(2A) receptor antagonist, istradefylline (KW-6002), in the forced swim test and the tail suspension test in rodents. *Pharmacol. Biochem. Behav.* 114-115 23–30. 24201052 10.1016/j.pbb.2013.10.022

[B63] Yoshimi K. Kaneko T. Voigt B. Mashimo T. (2014). Allele-specific genome editing and correction of disease-associated phenotypes in rats using the CRISPR-Cas platform. *Nat. Commun.* 5:4240. 10.1038/ncomms5240 24967838 PMC4083438

[B64] Yuan X. S. Wang L. Dong H. Qu W. M. Yang S. R. Cherasse Y. (2017). Striatal adenosine A2A receptor neurons control active-period sleep *via* parvalbumin neurons in external globus pallidus. *Elife* 6:e29055. 10.7554/eLife.29055 29022877 PMC5655138

[B65] Zhang J. P. Xu Q. Yuan X. S. Cherasse Y. Schiffmann S. N. de Kerchove A. (2013). Projections of nucleus accumbens adenosine A2A receptor neurons in the mouse brain and their implications in mediating sleep-wake regulation. *Front. Neuroanat.* 7:43. 10.3389/fnana.2013.00043 24409122 PMC3857888

